# MRI compatibility of the Autonomic Technologies Inc (ATI) SPG Neurostimulator – New Treatment for Cluster Headache (CH)

**DOI:** 10.1186/1129-2377-1-S14-P228

**Published:** 2013-02-21

**Authors:** R Powell, E Pless, A Caparso

**Affiliations:** 1 Autonomic Technologies, Inc., USA

## Introduction

The ATI SPG Neurostimulator is designed to be implanted in the mid-face anatomy and to electrically stimulate the SPG. The results from the multicenter European Pathway CH-1 study indicate that patient-controlled SPG stimulation provides statistically significant CH pain relief and is also associated with a reduction in CH frequency.

## Methods

MR safety testing was performed according to ASTM standards governing force, torque, image artifact, and RF heating. The SPG Neurostimulator has a mass of 1.5 grams, a thickness of less than 5mm, and is available in four lengths. All four lengths were used during RF heating and image artifact testing in both 1.5T and 3T MRI environments. Force and torque were measured using the longest Neurostimulator in a 3T environment, which represents the worst case scenario.

## Results

The maximum mean force generated (for both displacement and torque) was 6.7 grams-force, which is less than the mass of a 50 euro cent coin. Once anchored in the anatomy, the SPG Neurostimulator would require forces and torques an order of magnitude greater than those generated from the 3T MR field before displacement could occur. Diagnostic MR imaging near the implant location is affected by image artifacts extending 40mm from the SPG Neurostimulator, including the integral lead, with a spin and gradient echo pulse sequence. The shape of the distortion varied in all three planes, but the magnitude of distortion was similar. A temperature rise of 0.5°C and 2.4°C at a whole body average specific absorption rate (SAR) of ≥ 2 W/kg was measured at the SPG Neurostimulator in a 1.5T and 3T environment, respectively. The ATI SPG Neurostimulator has also been successfully imaged in multiple patients without any apparent side effects.

## Conclusions

Results from this testing has enabled the ATI SPG Neurostimulator to be labeled as MR conditional in the European Union, and is currently the only whole body MR conditional CE marked neurostimulator available.

## Conflict of interest

All authors are employees of Autonomic Technologies, Inc.

